# Sb(III) Removal by Granular Adsorbent Synthesized with Iron-Containing Water Treatment Residuals and Chitosan

**DOI:** 10.3390/polym16223214

**Published:** 2024-11-20

**Authors:** Huiping Zeng, Yuwei Zeng, He Xu, Siqi Sun, Jie Zhang, Dong Li

**Affiliations:** 1 Key Laboratory of Water Quality Science and Water Environment Recovery Engineering, Beijing University of Technology, Beijing 100124, China; 2 State Key Laboratory of Urban Water Resource and Environment, Harbin Institute of Technology, Harbin 150090, China

**Keywords:** antimony, adsorption, iron-containing water treatment residuals, chitosan, water treatment, granulation

## Abstract

In this study, chitosan and iron-containing water treatment residues were used to prepare a chitosan/Fe-sludge particle adsorbent (CHFS) via the embedding method for Sb(III) removal. Various technologies were applied to characterize the CHFS, and batch experiments were used to investigate its adsorption properties. The results show that CHFS adsorbents are amorphous and have a specific surface area (119.95 m^2^/g), both beneficial for adsorption. pH and ionic strength have no impact on the adsorption. Sb(III) adsorption on CHFS occurs spontaneously and endothermically. Sb(III) adsorption by CHFS matches the pseudo-second-order kinetic model and the Langmuir model better, with a maximum adsorption capacity of 24.38 mg/g. The primary adsorption mechanism for Sb(III) is the inner sphere complexation between the Sb and Fe–O bond, while other adsorption mechanisms include chelation, pore filling, and hydrogen bonding. This study offers a reference for antimony removal and resource utilization of iron sludge.

## 1. Introduction

Antimony, a highly toxic metallic element, primarily exists in natural waters in two forms, Sb(III) and Sb(V), with Sb(III) being significantly more toxic than Sb(V) [1]. Antimony concentrations in natural waters are typically kept below 1 mg/L [2]. However, industrial development—mainly using antimony in flame retardants, ceramics, glass, batteries, paints, and textiles—has led to the overexploitation of antimony ores. This has resulted in severe surface and groundwater contamination, with antimony levels rising dramatically to several mg/L and sometimes tens of mg/L [2,3,4,5]. High exposure to antimony poses serious health risks, including damage to the nervous system, liver, kidneys, and lungs, as well as potential genetic abnormalities and cancer [6]. Consequently, removing antimony from water sources, especially the more toxic Sb(III), has become an urgent concern.

Various removal methods have been developed to address this challenge, including coagulation and precipitation, membrane separation, ion exchange, electrochemical processes, and adsorption techniques [7,8,9,10]. Among these, adsorption is particularly favored due to its non-toxicity, high efficiency, ease of handling, and reusability [11,12]. Iron-based adsorbents have gained prominence in antimony removal studies owing to their large specific surface area, low cost, and predictable structure and function [13]. Most of these adsorbents come from chemically synthesized materials or iron-containing minerals, underscoring the need for sustainable alternatives in a low-carbon economy.

Recent research has increasingly focused on utilizing iron-based waste materials, such as textile sludge, magnetic sludge-derived biochar, and acid-treated iron-rich sludge, as antimony adsorbents, providing innovative avenues for development [14,15,16]. In northeastern China, iron-containing backwash sludge generated from iron and manganese removal plants has emerged as a promising raw material for iron-based adsorbents due to its high iron content [17]. However, practical applications of these sludge powders face challenges. While conventional powder adsorbents are efficient, they often suffer from loss, aggregation, and storage difficulties.

To address these issues, vacuum freeze-drying technology can transform hydrogels into pore-rich aerogels, enhancing the adsorption performance of the adsorbent. Aerogel beads offer higher adsorption capacity, greater specific surface area, and increased porosity as compared to powder forms. Their unique network structure provides more adsorption sites, while their optimal size helps mitigate mass loss, balancing economic viability with performance.

Chitosan, an amino polysaccharide, is widely used as a binder due to its environmental sustainability, cost-effectiveness, alkali resistance, and abundance of functional groups, such as hydroxyl and amino groups, which facilitate heavy metal adsorption [18]. These functional groups effectively adsorb various heavy metals, including Cu, Cr, As, and Sb [19,20,21,22]. Additionally, the positive charge of chitosan molecules enables them to bind to negatively charged metal–oxygen anions (e.g., Sb(III)) through electrostatic attraction [23], further enhancing adsorption capacity.

In this study, iron sludge was embedded in chitosan and made into chitosan/iron sludge microspheres (CHFS) as a novel antimony adsorbent using a vacuum freeze-drying technique. The effectiveness of CHFS for removing Sb(III) was verified through a detailed examination of the adsorption kinetics, isotherms, and the effects of pH, dosage, ionic strength, and coexisting ions on the adsorption effect. Characterization of CHFS was conducted using various methods, including SEM, BET, XRD, FTIR, and XPS, to investigate the adsorption mechanism of Sb(III) and the interactions between chitosan and iron sludge. This research promotes the resource utilization of waterworks sludge and proposes a new strategy for developing efficient and sustainable antimony adsorbents.

## 2. Materials and Methods

### 2.1. Material

Iron sludge was obtained from the backwash water of the ferromanganese removal biofilter of the Songbei Water Plant in Harbin, China. The Fe^2+^ concentration in the influent water of the water plant was 14.9 mg/L [24]. The supernate was dumped after the wastewater precipitated for some hours, and the yellowish-brown iron sludge collected on the bottom was left to dry naturally in the air. The iron sludge was thoroughly dried, ground passed through a 100-mesh sieve, and finally stored in a drying container for use. The iron sludge contained 89.04% Fe and small impurities, mainly Mn, Ca, K, and Si [24].

The sodium hydroxide (NaOH), hydrochloric acid (HCl), acetic acid (CH_3_COOH), thiourea (CH_4_N_2_S), and potassium borohydride (KBH_4_) used in this experiment were all analytically pure and purchased from Tianjin Fuchen Chemical Reagent Factory (Tianjin, China). To make Sb(III) solution, antimony potassium tartrate (C_8_H_4_K_2_O_12_Sb_2_∙3H_2_O) was employed. The chitosan had a medium viscosity (200–400 mpa∙s). McLean Biochemical Technology Co., Ltd. (Shanghai, China) provided both of these.

### 2.2. Adsorbent Preparation

**Figure 1:**
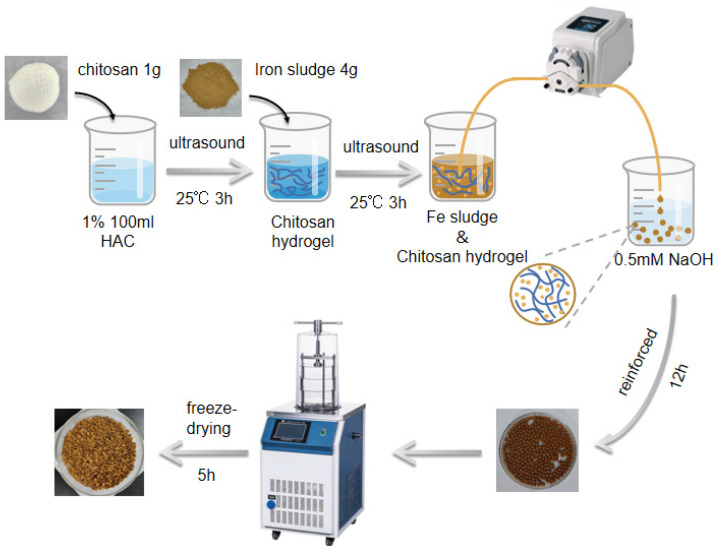
Schematic illustration of the synthesis of CHFS.

The embedding approach was used to create the adsorbent of chitosan/Fe-sludge particles (CHFS). A sufficient amount of iron sludge was essential to achieve optimal adsorption capacity, while an appropriate amount of chitosan ensured adequate mechanical strength and material morphology. After extensive laboratory testing, it was determined that the 1:4 ratio effectively balanced these factors, maximizing adsorption capacity while maintaining the necessary mechanical strength. Thus, a 1:4 ratio of chitosan to iron sludge was selected for granulation. The production process is shown in Figure 1. Firstly, 100 mL of 1% (*v*/*v*) acetic acid was mixed with 1 g of chitosan and then placed under ultrasound at room temperature for 3 h to produce the chitosan solution. After adding 4 g of iron sludge to the chitosan solution, the ultrasonic process continued for 3 h to ensure that the iron sludge was equally distributed. Then, the resting chitosan–iron sludge hydrogel was added to a 0.4 M sodium hydroxide solution to create gel beads using a soft tube peristaltic pump. The gel beads were reinforced in sodium hydroxide solution for 12 h to make the final adsorbent, repeatedly cleansed with deionized water, frozen at −55 °C for 12 h, and then dried in a freeze-dryer for 12 h [25].

### 2.3. Characterization

The surface appearance and size of CHFS were explored by scanning electron microscopy (SEM, Tecnai G2 F30, FEI, Hillsboro, OR, USA). The surface element distribution of CHFS was analyzed by elemental mapping of energy dispersive spectroscopy (EDS). Nitrogen adsorption/desorption isotherms of the samples were measured using an ASAP 2460 (Mack, Easton, PA, USA) absorptiometer. The Brunauer–Emmett–Teller (BET) and Barett–Joyner–Hallenda (BJH) methods were employed to estimate the surface area and pore size distribution. The composition of CHFS was determined using an X-ray diffractometer (XRD, Bruker D8 Advance, Bremen, Germany) with Co Kα radiation (l = 1.79026 A) conducted at a 2θ range of 10°~90°. The surface functional group shifts of CHFS before and after adsorption were analyzed by employing a Fourier transform infrared spectrometer (FTIR, Nicolet IS10, Nicolet, Madison, WI, USA) in the range of 500–4000 cm^−1^. X-ray photoelectron spectroscopy (XPS, Escalab 250Xi, Thermo Fisher Scientific, Waltham, MA, USA) was applied to analyze the valence states.

### 2.4. Batch Sorption Experiments

To make 1 g/L of Sb (III) stock solution, antimony potassium tartrate was dissolved in deionized water and stored in a 277.15 K refrigerator. When used, the stock solution was diluted to the needed concentration.

The batch experiments in this study were all performed by adding 40 mg of adsorbent to conical flasks containing 100 mL of antimony solution (pH = 6.6 ± 0.1), which will not be described below. The conical flasks containing the solution were set in a thermostatic orbit shaker and shaken at 165 rpm for 24 h. After a reaction, atomic fluorescence spectrophotometry was applied to quantify the antimony content in water samples.

In the adsorption kinetics experiment, 0.32 g adsorbent was added to 800 mL of 100 μg/L Sb (III) solution and water samples were taken at intervals of 0–40 h to determine the antimony content, and the pseudo-first-order kinetic model and pseudo-second-order kinetic model were used for fitting. In the isothermal adsorption experiment, the initial concentration of Sb ranged from 0.5 to 30 mg/L. The Langmuir model and Freundlich model were used to match the results at 298.15 K, 308.15 K, and 318.15 K. Adsorption isotherm tests on iron sludge and chitosan were also conducted under identical conditions at 298.15 K. To figure out the impact of pH, the initial pH of the solution was changed four to ten times using 0.1 M HCl and 0.1 M NaOH. 0.1 M KCl was used as the background electrolyte. The solutions were adjusted to different initial pH values (4–9) with 0.1 M HCl and 0.1 M NaOOH, and the final pH of each solution was determined after 24 h. The intersection of the final pH and initial pH curves corresponded to the zero-point charge of the adsorbent (pzc). The dose was established at 0.15 g/L–1.0 g/L in the dosage influence experiment, and the appropriate dosage was investigated. In the study of the influence of co-ionization on the adsorption effect, the effect of several common coexisting anions (Cl^−^, NO_3_^2−^, CO_3_^2−^, SO_4_^2−^, and PO_3_^4−^) on the adsorption effect in natural water was analyzed at levels of 0.01 mM, 0.05 mM, 0.1 M.

### 2.5. Adsorbent Regeneration and Reuse

Three consecutive adsorption and regeneration cycles were conducted to assess the reusability of CHFS. First, 40 mg CHFS was shaken in 100 mL Sb (III) for 24 h, and the saturated CHFS was separated from the solution. Then, it was shaken in 0.5 M 50 mL NaOH for 24 h to desorb Sb, and the concentration of Sb was measured by collecting the supernatant. The CHFS was constantly washed with deionized water until neutral after desorbing Sb. At this point, it was carried on to the next adsorption cycle.

### 2.6. Analysis of Sb

Atomic fluorescence spectrophotometry (AFS-8230, Beijing Jitian Instrument Co., Ltd., Beijing, China) was applied to measure the antimony content of the aqueous solution after the water that was collected was filtered using a 0.45 μm filter membrane. Equations (1) and (2) show how the Sb adsorption capacity of the CHFS and the rate of Sb removal were calculated.

(1) $$q_{e}=\left(C_{0}-C_{e}\right)V/m$$

(2) $$\%\;\mathrm{of}\;\mathrm{Remove}={(C}_{0}-C_{e})/C_{0}\times 100$$

where q_e_ (mg/g) is the capacity of adsorption at equilibrium, C_0_ and C_e_ (mg/L) are the concentration of antimony at the initial and final stage, V (L) is the reaction solution volume, and m (g) is the adsorbent dose.

## 3. Result and Discussion

### 3.1. Characterization of the Adsorbents

#### 3.1.1. SEM and EDS

**Figure 2:**
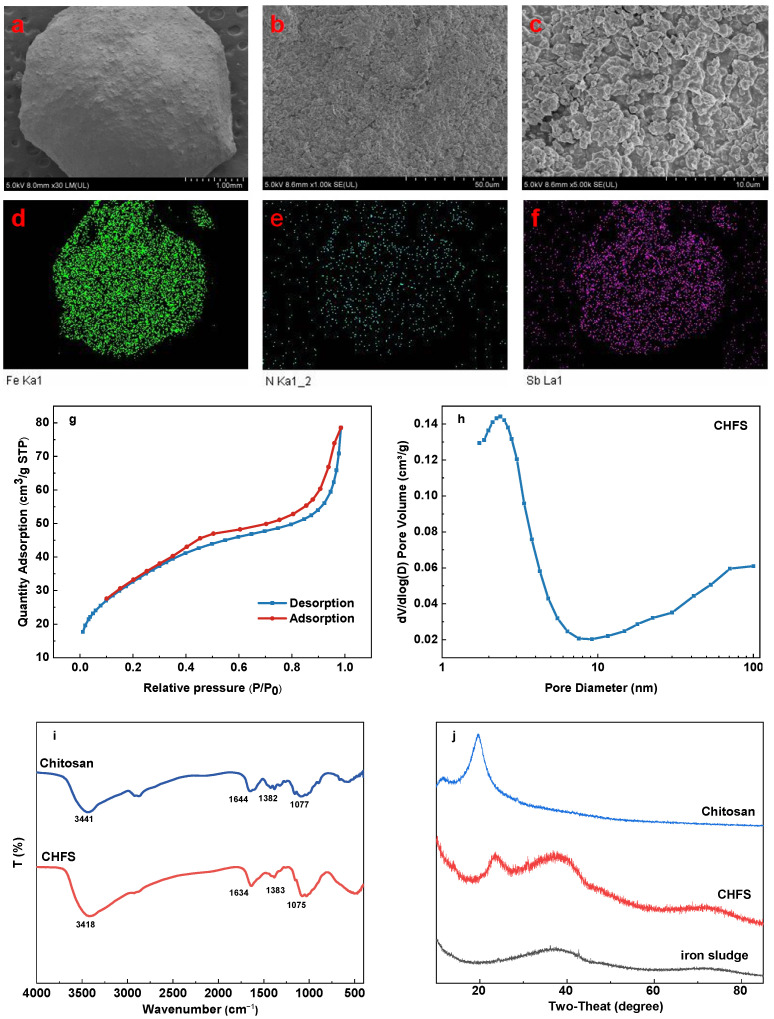
SEM of CHFS (**a**–**c**); EDS mapping analysis of CHFS: Fe (**d**), N (**e**), and adsorbed Sb (**f**); N_2_ adsorption-desorption isotherms of CHFS (**g**); pore size distribution of CHFS (**h**); FTIR spectra of CHFS and chitosan (**i**); XRD pattern of chitosan, CHFS, and iron sludge (**j**).

The SEM results for CHFS are shown in Figure 2a–c. The image demonstrates that CHFS is a spherical particle adsorbent with a diameter of roughly 3 mm. The surface of CHFS has been folded, seems rougher, and is formed of medium and large pores with a rich, layered porous structure. These surface characteristics support raising the specific surface area of CHFS and make it easier for Sb(III) to diffuse into the particles, which promotes the adsorption of Sb(III).

The energy dispersive spectroscopy (EDS) mapped scan pictures of CHFS are shown in Figure 2d–f. The image shows the inclusion of N, Fe, and adsorbed Sb in CHFS. The majority of the N element and the majority of the Fe element in CHFS come from chitosan and iron sludge, respectively. The interaction of chitosan and iron sludge can also evidence this.

#### 3.1.2. BET

Figure 2g illustrates that, according to IUPAC classification, the N_2_ adsorption-desorption isotherm is classified as type IV with an H_3_ hysteresis loop. This indicates the significant existence of mesoporous structures in CHFS [26]. Notably, the CHFS sample displays an extended hysteresis loop in the P/P_0_ Scholarship range of 0.4–1.0, which can be attributed to capillary condensation within the pores. This observation further confirms the abundance of mesopores in the sample [17]. The aperture distribution curve in Figure 2h indicates a predominance of mesopores (2 nm < d < 50 nm), with some macropores (d > 50 nm) present [27]. These results are consistent with TEM observations. While macropores facilitate liquid transfer, micropores and mesopores enhance the effective contact area with Sb (III) [28]. The BET showed a specific surface area of 119.95 m^2^/g, which favors the efficient adsorption of Sb (III).

#### 3.1.3. FTIR

Figure 2i shows the FTIR of chitosan and CHFS. The peak produced by the stretching vibration overlap of the N–H and O–H bonds in chitosan moved from 3441 cm^−1^ to 3418 cm^−1^.

After CHFS was prepared using the embedding approach, the peak breadth increased. This may be related to the redshift created by intermolecular hydrogen bonds between the –OH in the iron sludge and the –OH or –NH in the chitosan, which leads to the multi-molecular association and broadens its peak. The absorption peaks of CHFS at 1634 cm^−1^, 1383 cm^−1^, and 1075 cm^−1^ are related to the stretching vibration of C–O, the symmetric angular deformation of –CH_3_, and the stretching and vibration of C–O from β(1→4) glycosidic, respectively, which all belong to the functional groups of chitosan [29,30]. These demonstrated that iron sludge and chitosan were successfully combined.

#### 3.1.4. XRD

Figure 2j shows the iron sludge, chitosan, and CHFS XRD patterns. The graph shows that chitosan has pronounced broad peaks at 11° and 20°, which belong to crystal types I and II of chitosan, respectively [31]. The X-ray particle diffraction pattern of iron sludge has a wide peak at around 38 degrees. The broad peaks at 23° and 38° in the XRD of CHFS are similar to those of chitosan and iron sludge, respectively. This also indicates the successful combination of chitosan and iron sludge. Because of the formation of intermolecular hydrogen bonds between the iron sludge and the chitosan, the chitosan-related peak increased from 20° to 23°. Additionally, the XRD of CHFS revealed no clear distinctive peaks, indicating that its structure is amorphous, which is more beneficial for adsorption.

### 3.2. Batch Sorption Experiments

#### 3.2.1. Effect of Adsorbent Dosage

**Figure 3:**
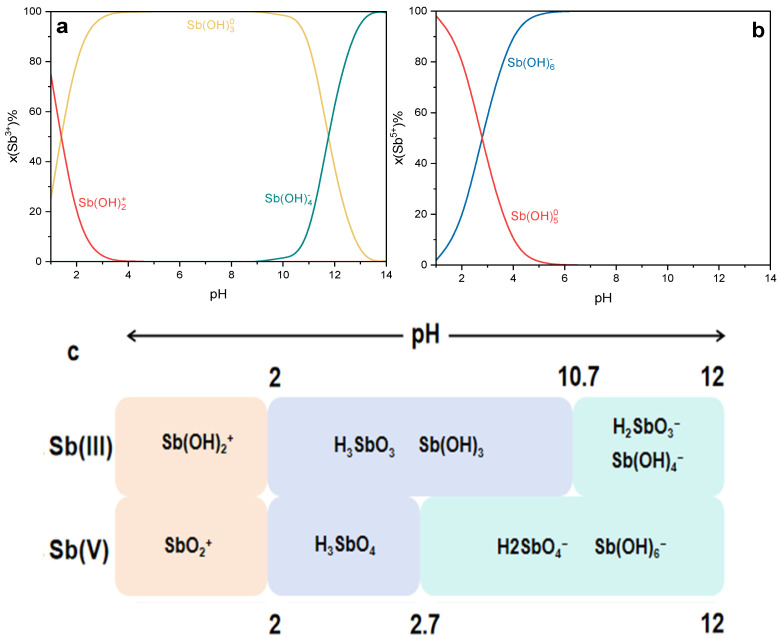
Ionic fractionation diagrams for the hydrolysis of Sb(III) (**a**) and Sb(V) (**b**) in water, and primary chemical speciation (**c**) of Sb in water.

The impact of the adsorbent dosage on Sb(III) sorption is shown in Figure 3a. The graph indicates that the removal rate of Sb(III) grew as the dosage was increased, and when the dose of adsorbent exceeded 0.4 g/L, the change in removal rate tended to remain steady and reached more than 90%. However, the adsorption capacity kept decreasing. This was because the dosage growth may have boosted the number of active adsorption sites to remove Sb(III) on the adsorbent surface. At the same time, the mass-to-weight ratio of Sb(III) and CHFS continued declining, which rendered it difficult to effectively utilize the adsorption sites on its surface, causing an increase in adsorbent dosage worthless for Sb(III) removal. Therefore, a suitable dose should be selected for the removal of antimony. For the following study, the optimal amount of CHFS was determined as 0.4 g/L.

#### 3.2.2. Effect of pH and Ionic Strength

The pH of the solution influences the speciation of Sb and the surface charge of the adsorbent, which in turn affects the electrostatic attraction between antimony compounds and adsorbents [32]. Therefore, pH is one of the main variables influencing the Sb(III) adsorption effect.

The distribution morphology of Sb(III) and Sb(V) in water at various pH levels was computationally simulated using Visual MINTEQ version 3.1, as illustrated in Figure 3a,b. Previous studies have examined the hydrolysis reactions of antimony in both the Sb(III)-H_2_O and Sb(V)-H_2_O systems, with the primary morphological forms detailed in Figure 3c [33,34].

In the Sb(III)-H_2_O system, antimony primarily exists as Sb(OH)_3_ (or SbO(OH), HSbO_2_) and can decompose under certain conditions to form colloidal Sb_2_O_3_, an insoluble substance. The hydrolysis reaction for Sb(OH)_3_ can be summarized as follows:

$${Sb(OH)}_{3}+yH_{2}O={Sb(OH)}_{3+y}^{y^{-}}+yH^{+}$$

Under acidic conditions (pH < 2), the hydrolysis products are predominantly in the form of ${Sb(OH)}_{2}^{+}$ (y = 1) or $S{bO}^{+}$. Conversely, under alkaline conditions (pH > 12), the hydrolysis products are predominantly in the form of ${Sb(OH)}_{4}^{-}$ (y = 1) or ${SbO}_{2}^{-}$. In weakly acidic to neutral conditions (pH 2–10), antimony typically exists as Sb(OH)_3_ or SbO(OH), while the divalent and trivalent cationic forms of Sb(III) are less commonly detected.

In the Sb(V)–H_2_O system, Sb(V) is mainly present as a solid monobasic acid, known as antimony acid, with the chemical formula H[Sb(OH)_6_] (or Sb(OH)_5_, HSbO_3_). The hydrolysis reaction for Sb(V) can be expressed as follows:

$${xSb(OH)}_{5}+yH_{2}O={Sb_{x}(OH)}_{5}^{x}+yH^{+}$$

**Figure 4:**
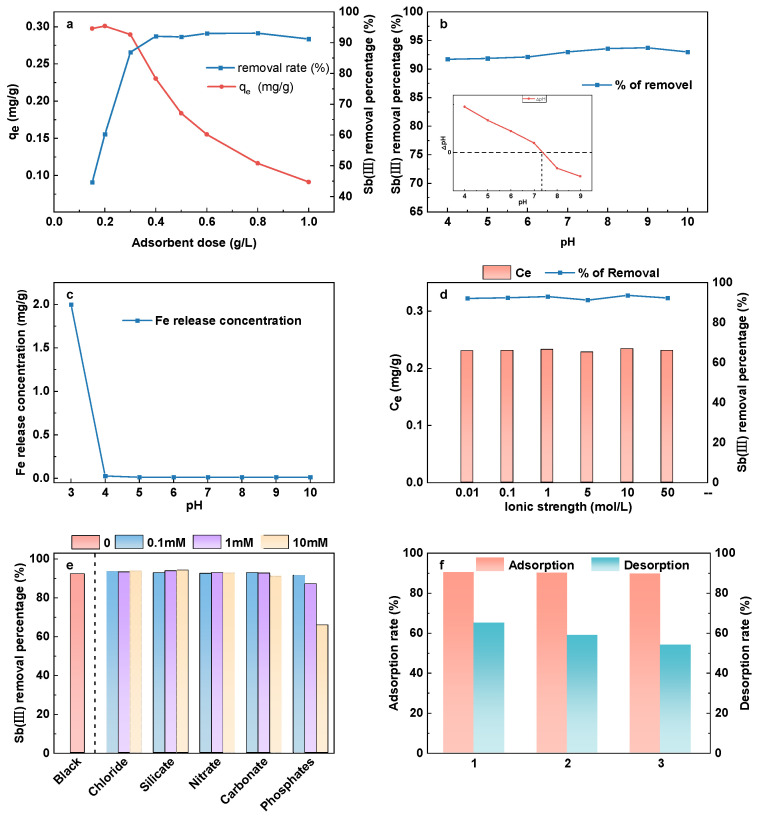
Effect of dosage of CHFS (**a**); effect of initial solution pH (pH = 4–10), inset: pH_pzc_ determination curve (**b**); dissolution of iron at different pH (pH = 3–10) (**c**); impact of ionic strength (**d**); effect of coexisting anions (**e**); the adsorption and desorption rate of Sb(III) by CHFS in 3 consecutive cycles (**f**).

In this experiment, the initial pH was altered between 4 and 10, and Figure 4b displayed that the removal rate of Sb(III) was above 90% during the whole pH range applied to the experiment. The adsorption effect of Sb(III) was unaffected by pH in a substantial way. The finding was similar to previous studies on antimony adsorption by iron-based material [35,36].

The majority of Sb(III) existed in solution as uncharged Sb(OH)_3_ in the whole pH range of this study (pH = 4–10), as indicated by the speciation scheme presented in Figure 3c. The pH_pzc_ of CHFS is about 7.5, as shown in Figure 4b. When the pH of the solution was less than pH_pzc_, the CHFS surface was positively charged, and vice versa. The adsorption effect, however, is not pH-dependent, indicating that electrostatic attraction is not the primary adsorption mechanism. Moreover, the adsorption impact does not significantly vary when the ionic strength is between 0.01 and 50 mM, according to the findings of the ionic strength experiment (Figure 4c). Therefore, it may be assumed that CHFS and Sb form an inner sphere complex to achieve the removal goal [36,37].

Figure 4d depicts the measurement of the concentration of leached iron in solution after the adsorption of Sb by CHFS at various solution pH levels (3–10). The Fe release concentration was not substantial (below the detection limit) at different pH levels, other than in the highly acidic environment (pH = 3), demonstrating the excellent stability of CHFS.

#### 3.2.3. Effect of Coexisting Anions

Antimonates frequently occur in natural water with anions such as chloride, nitrate, sulfate, carbonate, silicate, and phosphate. As a result, this study investigates how these ions influence the adsorption impact of Sb(III) in different concentrations (0.1 mM, 1 mM, and 10 mM), and the outcomes are displayed in Figure 4e.

Figure 4e indicates that chloride, carbonate, sulfate, and nitrate had no discernible effect on the adsorption of Sb, while phosphate had an apparent impact. The initial removal rate of 93% decreased to 86% when the phosphate concentration was at 1 mM and 65% at 10 mM.

Bonding interactions could be a way of clarifying why these coexisting ions had different effects. Chlorides and nitrates combine with iron oxides to form exospheric complexes [38]. Sulfate also forms an exosphere complex when pH exceeds 6 [39,40]. These three ions had little impact on the adsorption because antimony and iron oxide combined to form a more stable inner sphere complex. Carbonate could also form the inner sphere complex; however, because of its poor affinity, the adsorption influence could be ignored [41]. Phosphates, on the other hand, created stronger inner sphere complexes that competed with antimony for adsorption sites, affecting the adsorption effect of CHFS on Sb [42].

#### 3.2.4. Adsorbent Regeneration and Reuse

Alkaline circumstances are thought to be appropriate for desorption. By way of ion exchange, deprotonation, electrostatic contact, and other processes, strong bases like NaOH can desorb Sb [43]. As a result, NaOH was selected as the desorption agent in this paper. As shown in Figure 4f, the adsorption rate did not drop drastically after three cycles, staying at about 90%. The desorption rate dropped as cycles increased, but remained at roughly 60%. The outcomes demonstrated the possibility of reusing the regenerated CHFS.

### 3.3. Adsorption Kinetics

**Figure 5:**
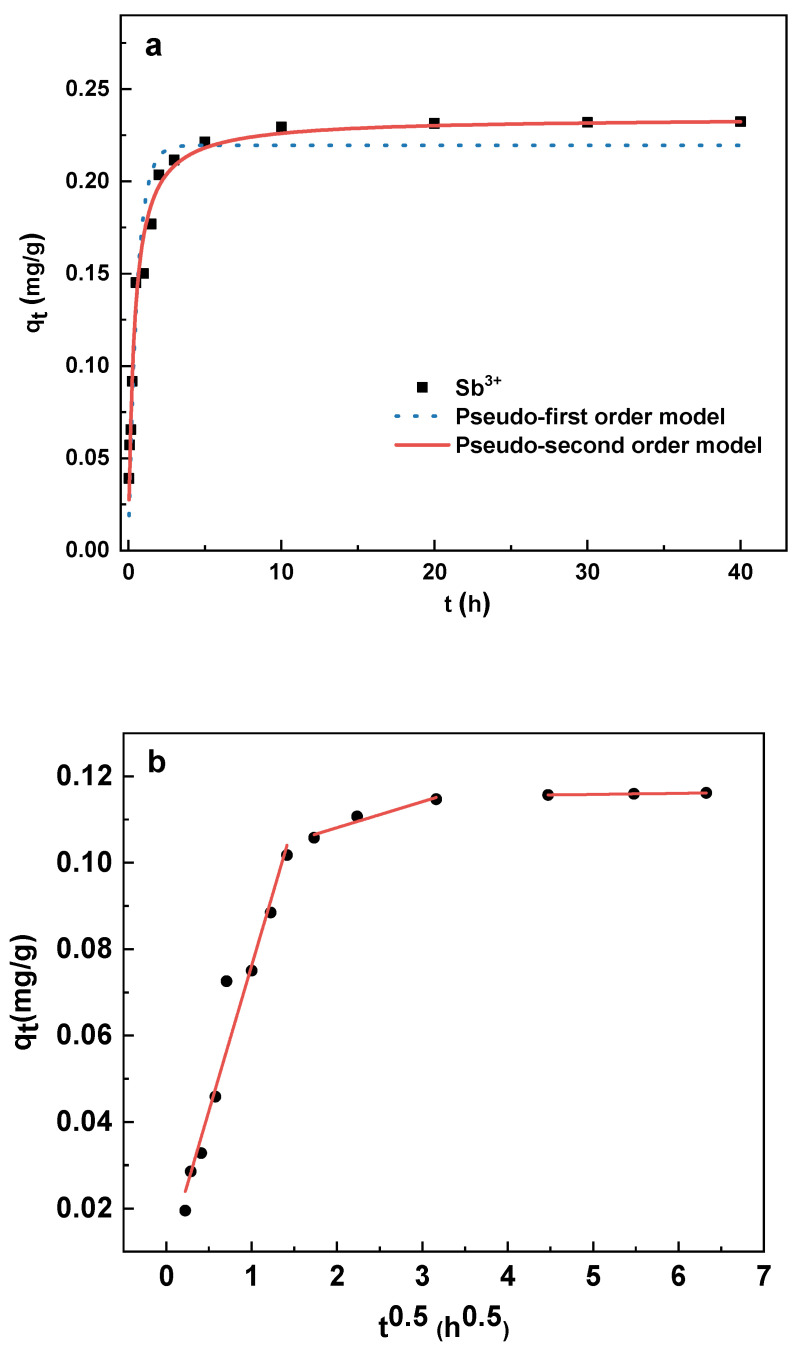
Adsorption kinetics of Sb(III) on CHFS (298.15 K pH 6.6 ± 0.1) (**a**); intra-particle diffusion model (298.15 K pH 6.6 ± 0.1) (**b**).

The results of this experiment, which analyzed the Sb(III) adsorption capacity of CHFS with time, are shown in Figure 5a. Due to the high Sb(III) level in the solution and the abundance of early-stage adsorption sites in the adsorbent, the Sb(III) adsorption rate was faster in the first 5 h of CHFS exposure. The adsorption site steadily became saturated with time, the Sb(III) level in the solution progressively decreased, and the adsorption rate gradually slowed. After ten hours, the adsorption achieved equilibrium, and the absorption rate reached 90%. The removal rate exceeded the 80% efficiency achieved for Sb (III) in reservoir water using PFS as a coagulant [44].

The pseudo-first-order model (Equation (3)) and pseudo-second-order model (Equation (4)) were fitted to match the data to offer a more accurate description of the adsorption kinetics. The pseudo-first-order kinetic model mainly describes the physical adsorption, which involves forces like hydrogen bonds and van der Waals interactions. The equation of pseudo-first-order dynamics is as follows:

(3) $$q_{t}=q_{e}\left(1-e^{-k_{1}t}\right)$$

The pseudo-second-order model primarily represented chemisorption, which involves exchanging and sharing electrons. The pseudo-second-order model is described by Equation (2).

(4) $$q_{t}={\left(1/q_{e}+1/k_{2}q_{e}^{2}\right)}^{-1}$$

where q_t_ (mg/g) and q_e_ (mg/g) represent the adsorption amount of Sb(III) at t time and equilibrium time on the unit adsorbent, respectively, and k_1_ (h^−1^) and k_2_ (g/(mg∙h) are the pseudo-first-order and pseudo-second-order kinetic constants, respectively. The fitting was carried out via non-linear regression using Origin version 2021.

Table 1 shows the adsorption kinetics fitting parameters. The findings indicate that the pseudo-second-order model (r^2^ = 0.983) has a higher correlation coefficient than the pseudo-first-order model (r^2^ = 0.939), suggesting that the former is more suitable to describe the adsorption of Sb(III) by CHFS.

The diffusion rate influences the adsorption of adsorbate in porous materials. Figure 5b displays the results of fitting the data with the Weber–Morris intra-particle diffusion model (Equation (5)), which was employed in this study to clarify the diffusion mechanism of the adsorption process.

(5) $$q_{t}=k_{i}t^{0.5}+C_{i}$$

where k_i_ is the intra-particle diffusion rate constant of stage I, and C_i_ is the constant associated with the thickness of the boundary layer.

Intra-particle diffusion was not the only rate-limiting process, as proven by the fitted values in Table 1, where the intercept was not zero (C_i_ ≠ 0). Multiple steps controlled the adsorption process. There were three steps to the Sb(III) adsorption by CHFS, as shown in Figure 5b. Film diffusion was the first step. In this step, Sb(III) was transferred from the solution to the outer surface of CHFS, where an adsorption site absorbed it. The gradual adsorption step based on intraparticle diffusion was the second stage. Sb(III) progressively diffused from the adsorption site on the outer surface to the adsorption site on the inner surface of the CHFS in this stage. The equilibrium stage was the last. At this stage, the adsorption sites on the inner and outer surfaces of the adsorbent were gradually saturated, and an equilibrium was obtained.

### 3.4. Adsorption Isotherms

**Figure 6:**
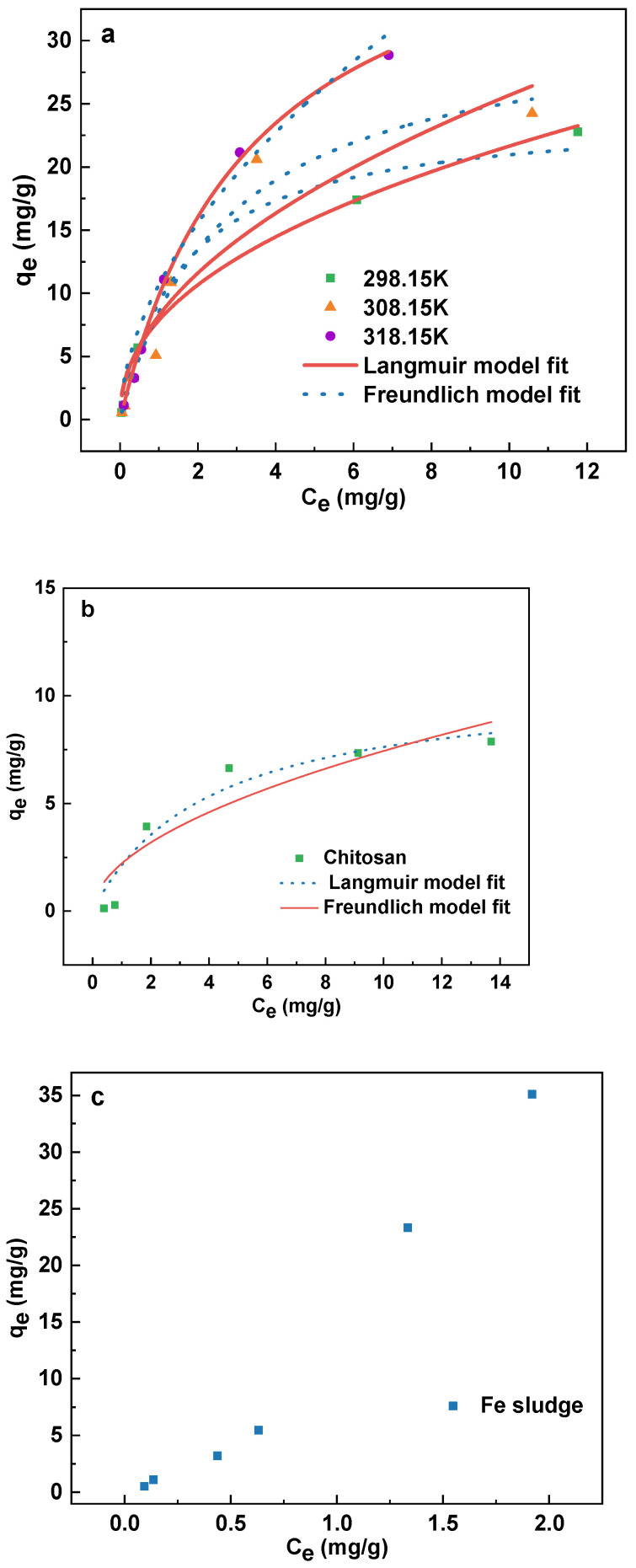

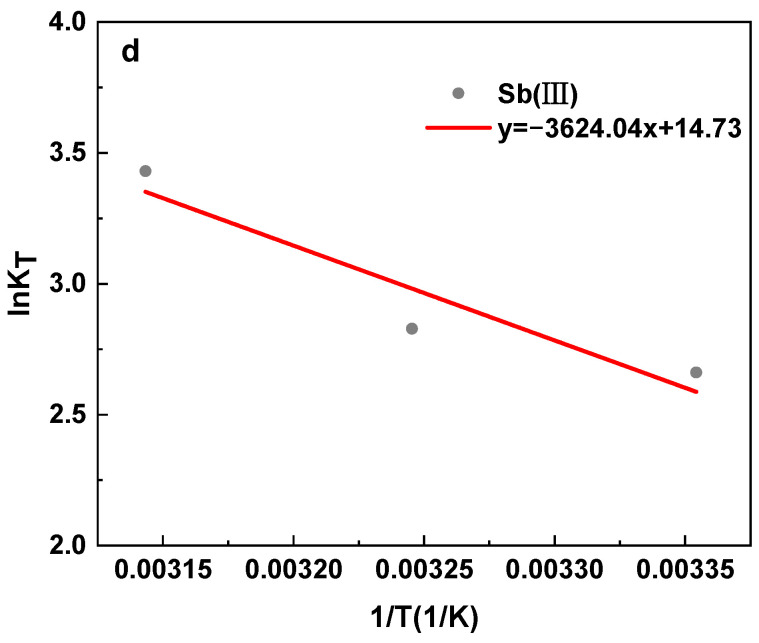
Langmuir and Freundlich isotherm models for Sb(III) adsorption of CHFS (298.15 K, 308.15 K, and 318.15 K at pH 6.6 ± 0.1) (**a**), chitosan (298.15 K pH 6.6 ± 0.1) (**b**), adsorption isotherm for Sb(III) adsorption of iron sludge (**c**), and the Van’t Hoff plots for Sb(III) adsorption of CHFS (**d**).

The isothermal adsorption experiment serves as an aid in clarifying the adsorption procedure in greater detail and determining the maximum adsorption capacity of the adsorbent. Therefore, isothermal adsorption studies of Sb(III) by CHFS were performed in this study at three different temperatures (298.15 K, 308.15 K, and 318.25 K), and isothermal adsorption models by Langmuir (Equation (6)) and Freundlich (Equation (8)) were applied to match the experimental results. Figure 6a shows the fitted adsorption isotherm. Table 2 displays the relevant model parameters which have been fitted.

The Langmuir model is suitable for describing monolayer adsorption occurring on a uniform surface, and its formula is as follows:

(6) $$q_{e}=q_{m}K_{L}c_{e}/\left(1+K_{L}c_{e}\right)$$

where c_e_ (mg/L) is the equilibrium concentration of Sb(III) in solution, q_e_ (mg/L) is the amount of Sb(III) that can adsorb on the adsorbent per unit mass when it is in equilibrium, q_m_ is the monolayer saturated adsorption of adsorbent capacity per unit weight, and K_L_ (L/mg) is the Langmuir affinity constant, which is correlated with the adsorption energy. The formula for the separation factor R_L_, a dimensionless adsorption parameter of the adsorbent to adsorbed material, is as follows:

(7) $$R_{L}=1/\left(1+K_{L}C_{0}\right)$$

where C_0_ is the greater beginning concentration in this instance. When R_L_ = 0, it means that the adsorption process is irreversible; when 0 < R_L_ < 1, it means that the adsorption process is ideal; when R_L_ = 1, it means that the adsorption process is linear; and when R_L_ > 1, it means that the adsorption process is unfavorable.

The Freundlich model is appropriate for describing multilayer adsorption on heterogeneous surfaces. The formula runs as follows:

(8) $$q_{e}=K_{F}c_{e}^{1/n}$$

K_F_ is the Freundlich adsorption constant, and n is the heterogeneous factor. 1/n may also be used as a favorable indication of adsorption. When its value is between 0 and 1, adsorption is simple to occur, and when its value is larger than 2, adsorption is complex to occur [45].

The fitting results reveal that the two fitting lines’ regression coefficients (R^2^) are more than 0.9, which shows that the two models match the data satisfactorily. The R^2^ of the Langmuir model is higher than that of the Freundlich model at different temperatures, proving that the adsorption of Sb(III) by CHFS is more suitable with the Langmuir model and that the majority of the adsorption on CHFS is monolayer adsorption. The values of R_L_ at different temperatures were all in the range of 0 to 1, demonstrating that Sb(III) was effectively adsorbable by CHFS. The fitting outcomes of the Freundlich model further support this. Table 2 shows that in the temperature range experiment, 1/n is close to 0.5, indicating that the adsorption of Sb(III) by CHFS is a reasonably easy action. The fitted isothermal experiment findings further support the viability of CHFS for Sb(III) removal.

The Langmuir model is capable of fitting the maximal adsorption capacity. The maximum adsorption capacity of CHFS for Sb(III) at 298.15 K, 308.15 K, and 318.25 K is 24.38 mg/g, 31.97 mg/g, and 43.69 mg/g, respectively, according to the fitting outcomes. The adsorption capacity increases with rising temperature, either because the adsorption process is an endothermic reaction or possibly because a boost in temperature accelerates the movement of Sb(III), increasing the chance that Sb(III) will come into contact with CHFS.

Additionally, studies involving the isothermal adsorption of Sb(III) by chitosan and iron sludge were carried out at 298.15 K, and outcomes are depicted in the attached Figure 6b. The relevant fitting data are displayed in Table 2. The findings demonstrate that the R^2^ of the Langmuir model is more significant than that of the Freundlich model and that most of the adsorption of Sb(III) by chitosan occurs in a monolayer. However, due to CHFS having a bigger K_L_ (0.61 L/mg) than chitosan (0.25 L/mg), it also has a stronger affinity for Sb(III) than chitosan does. Chitosan and CHFS displayed maximal adsorption capacities of 10.69 mg/g and 24.38 mg/g, respectively, at 298.15 K, proving that CHFS has more affinity for Sb than chitosan. Figure 6c exhibits the isothermal adsorption outcomes of iron sludge at 298.15 K. Langmuir and Freundlich models cannot match the iron sludge isothermal adsorption data. The equilibrium adsorption capacity of Sb(III) in the experiment reached 35.1 mg/g, significantly more than the adsorption saturation capacity of chitosan when the initial Sb(III) level was 30 mg/L. Thus, it can be shown that iron sludge performs the primary adsorption function in CHFS, whereas chitosan contributes to adsorption weakly, and mainly acts to support iron sludge.

The saturation capacity of CHFS is lower than that of iron sludge because (1) the process of chitosan embedding may make it more challenging to expose part of the adsorption sites of the iron sludge, and (2) iron sludge is more diffusible in solution as powder. Table 3 lists the Sb(III) adsorption saturation capacities for multiple iron-based, carbon-based, and biological adsorbents. Although CHFS has a lower adsorption saturation capacity than some iron-based materials, it is simple to create, applies greener preparation materials, is cheaper, and has a greater adsorption saturation capacity than carbon-based and biological adsorbents. Because of this, CHFS emerges as a promising adsorbent in removing antimony from water.

### 3.5. Thermodynamic Model

Adsorption thermodynamics is a standard method to evaluate thermodynamic energy and spontaneity in adsorption. The thermodynamic parameters, such as Gibbs free energy ∆G^0^ (kJ/mol), standard enthalpy change ∆H^0^ (kJ/mol), and entropy change ∆S^0^ (kJ/(mol∙K)), are calculated using Equations (9)–(12):

(9) $$K_{T}=q_{e}/c_{e}$$

(10) $${\Delta G}^{0}={-RTlnK}_{T}$$

(11) $${\Delta G}^{0}={\Delta H}^{0}-{T\Delta S}^{0}$$

(12) $${lnK}_{T}={\Delta S}^{0}/R-{\Delta H}^{0}/RT$$

where T is the temperature (K), K_T_ is the thermodynamic equilibrium constant (L/g), and R is the general gas constant, with a value of 8.314 × 10^−3^ kJ/(mol∙k).

The van’t Hoff plots (Figure 6d) were plotted with 1/T and lnKT as the horizontal and vertical coordinates, and ΔH^0^ and ΔS^0^ were calculated from the slope and the intercept. The relevant thermodynamic parameters are listed in Table 4. The thermodynamic parameters ΔH^0^ and ΔS^0^ were calculated from the slope and intercept of these plots, with the results summarized in Table 4. Notably, the calculated values of ΔG^0^ were −6.597 kJ/mol, −7.248 kJ/mol, and −9.047 kJ/mol at 298.15 K, 309.15 K, and 319.15 K, respectively. All ΔG^0^ values were negative and exhibited a decreasing trend with increasing temperature, indicating that the adsorption of CHFS on Sb(III) is spontaneous and thermodynamically favorable as the temperature rises.

Furthermore, ΔH^0^ was greater than 0, confirming the endothermic nature of the adsorption process. According to [31], adsorption can be categorized based on the absolute value of ΔH^0^: values between 0 and 20 kJ/mol indicate physical adsorption, while those between 80 and 400 kJ/mol suggest chemical adsorption. Our calculated ΔH^0^, falling between 20 and 80 kJ/mol, implies that both physisorption and chemisorption contribute to the adsorption of Sb(III) by CHFS.

Additionally, Table 4 indicates that ΔS_0_ is positive. This aligns with the expectation that inner-sphere complex formation typically results in positive ΔS, as it can displace solvated water molecules at the adsorption site, unlike outer-sphere complexes [54,55,56]. The endothermic nature of chemisorption provides a logical explanation for observing ΔS > 0.

### 3.6. Sorption Mechanism

Since the adsorption process is unaffected by pH, as shown by the outcome of the previous batch experiment, electrostatic attraction is not the primary adsorption mechanism. A more stable inner sphere complex seems to form because the effect of adsorption on ionic strength and pH is relatively minimal.

**Figure 7:**
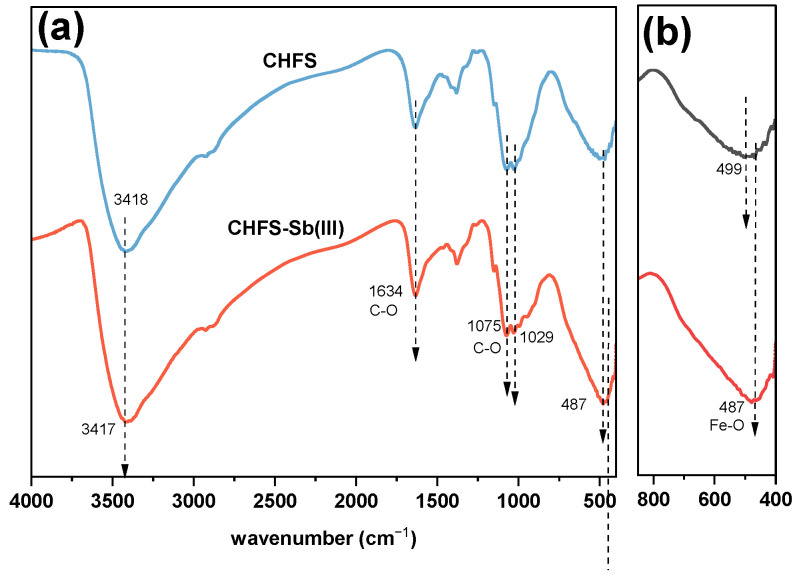
(**a**) FTIR of CHFS before and after adsorption of Sb(III) (**b**) Local magnified FTIR.

The FTIR plot is shown in Figure 7, before and after CHFS has adsorbed antimony. After adsorption of Sb(III), the iron on the surface of the adsorbent may oxidize and come into contact with air, resulting in a broadening of the peak of the Fe–O group at the peak at 487 cm^−1^ [57]. In addition, the peak of the Fe–O bond shifted slightly from 499 cm^−1^ to 487 cm^−1^, which may be attributed to the formation of the Fe–O–Sb bond. The results suggest that Fe–O bonds are essential in removing antimony [58].

**Figure 8:**
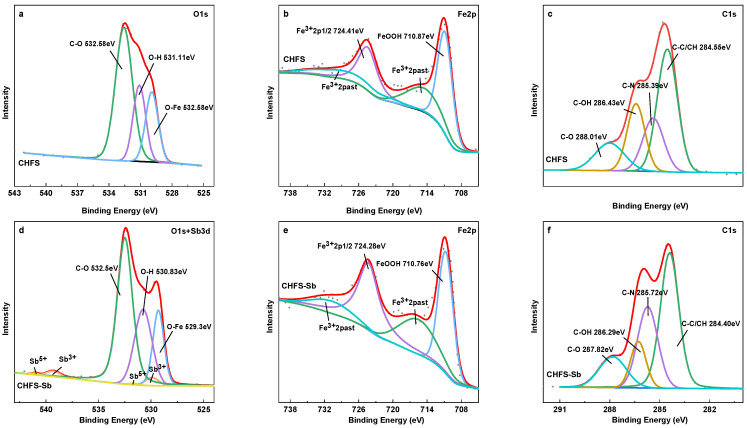
The spectra of O1 s (**a**,**d**); Fe2p (**b**,**e**); and C1s (**c**,**f**) before and after Sb(III) adsorption.

The XPS scanning pattern is shown in Figure 8. Figure 8a shows a narrow spectrum scan of O1s. The location of the Sb3d_5/2_ front coincides with the O1s peak. Sb3d_3/2_ may be further subdivided into 539.29 ev and 540.01 ev, belonging to Sb^3+^ and Sb^5+^, respectively, based on the peak sub-fitting findings of the narrow spectrum scan of O1s+Sb3d (Figure 8d) [22,59]. Therefore, it can be inferred that CHFS efficiently adsorbs Sb and that a minority of Sb(III) was oxidized to Sb(V), which is compatible with the FTIR data. Sb(III) has a low Eh and a more straightforward oxidizing phase. The particular reasons might be as follows: (1) the adsorbed CHFS is in contact with the air, which causes the Sb on its surface to be oxidized [37]; or (2) the coordination of Sb(III) with Fe increases the electron density of Sb atoms, which in turn promotes the oxidation process [35].

The Fe2p is depicted in Figure 8b,d before and after Sb(III) adsorption by CHFS. The fitting data demonstrate that the iron in CHFS is amorphous FeOOH, compatible with the above XRD findings. After adsorption, the binding energies of Fe2p_3/2_ and Fe2p_1/2_ dropped from 710.87 eV and 724.41 eV to 710.70 eV and 724.28 eV, respectively. This might have resulted from the electron transfer that occurs when Fe and Sb form complexes, which lowers the binding energy. Based on the O1s narrow spectrum scan of CHFS (Figure 8a,d), the binding energy of the O–Fe bond reduced from 529.92 eV to 529.30 eV after adsorption, and the integral area ratio decreased from 21.02% to 19.58%. All of them demonstrated that Fe was involved in the process of Sb adsorption to create the Fe–O–Sb inner-sphere complex. Potential chemical processes might result in the inner-sphere complex when Sb(III) and FeOH interact.

≡FeOH + Sb(OH)_3_ + H^+^→≡FeSb(OH)_3_ ^+^ + H_2_O

≡FeOH + Sb(OH)_3_→≡FeOSb(OH)_2_ + H_2_O

≡FeOH + Sb(OH)_3_→≡FeOSbO(OH)^−^ + H_2_O + H^+^

≡(FeOH)_2_ + Sb(OH)_3_ + 2H^+^→≡(Fe)_2_Sb(OH)_3_^2+^ + 2H_2_O

Additionally, the binding energy of C–N and C–OH shifted following adsorption, as demonstrated by the C1s narrow spectrum scan of CHFS (Figure 8c,f). This could be due to hydrogen bonds forming between –OH and Sb and chelation between –NH_2_ and Sb in chitosan. O1s showed an –OH shift from 531.11 eV to 530.83 eV. This finding coincides with that of FTIR spectroscopy, which shows that following adsorption, –OH/–NH is redshifted from 3418 cm^−1^ to 3417 cm^−1^. This indicates that –OH and –NH are also the binding sites for Sb in chitosan [60].

**Figure 9:**
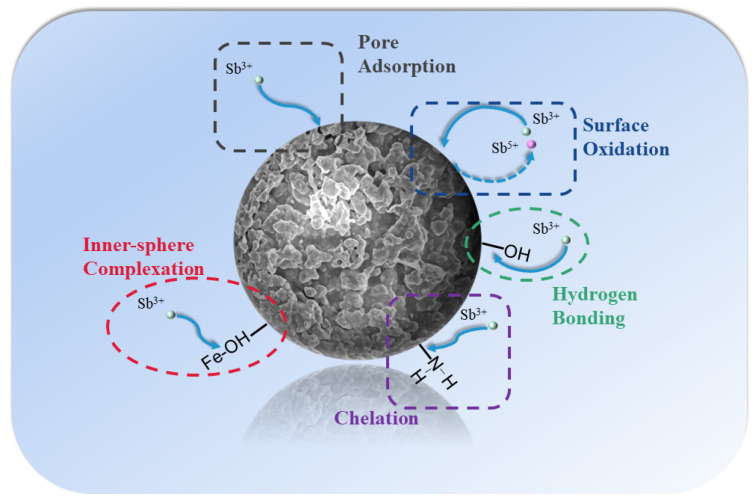
The mechanisms of Sb(III) sorption by CHFS.

In summary, Figure 9 shows the Sb(III) adsorption process by CHFS. The abundance of pores in CHFS, which play a part in adsorption and serve as Sb(III) diffusion channels, results from freeze-drying. The CHFS comprises chitosan and iron sludge, where the Fe–OH of the iron sludge coordinates with Sb to form an inner sphere complex, the –NH_2_ of the chitosan chelates Sb, and the hydrogen bonds that are created between the –OH and Sb produce the removal effect. According to the adsorption saturation capacity in the isotherm, the iron sludge plays the predominant adsorption function, since the adsorption saturation capacity of chitosan for Sb is only 6 mg/g. In CHFS, a small amount of Sb(III) was also oxidized to Sb(V).

## 4. Conclusions

CHFS was prepared to absorb antimony in water by backwashing iron sludge with chitosan embedding. According to the characterization results, CHFS is amorphous and contains plenty of pores, which are both advantageous for Sb adsorption. Chitosan hydrogel adhesion and an intermolecular hydrogen bond link chitosan and iron sludge together. The outcomes of batch experiments demonstrate that the pseudo-second-order model better predicts the Sb(III) adsorption by CHFS. Using Freundlich and Langmuir models when describing the adsorption process is appropriate. The maximum adsorption saturation capacity of CHFS for Sb(III) was 24.38 mg/g, according to the Langmuir model. Chloride ions, sulfate, silicate, and nitrate had little impact on the adsorption, but phosphoric acid did. The CHFS adsorption action was still strong after three cycles. It is believed that electrostatic adsorption is not the primary adsorption mechanism for Sb(III), and CHFS forms an inner sphere complex with Sb(III) since the adsorption impact of Sb(III) is nearly entirely unaffected by pH and ionic strength. The process for the adsorption of Sb by CHFS is as noticed in the XPS diagram before and after adsorption. Following the adsorption of Sb on CHFS, some Sb(III) was converted to Sb(V), and Fe and Sb interacted to form an inner sphere complex, the primary adsorption mechanism. In addition, Sb was also bound by the –NH and –OH of chitosan.

Antimony adsorption in water may profit from using CHFS, a cheap, environmentally beneficial, and pollution-free adsorbent. However, further study on the adsorbent’s performance in the continuous mode of the adsorption column must be performed to assess its appropriateness in real-world scenarios.

## Figures and Tables

**Table 1:** Adsorption kinetic parameters and Weber–Morris particle pore diffusion model parameters.

**Pseudo-first-order**			**Pseudo-second-order**		
q_e_ (mg/g)	k_1_ (h^−1^)	r^2^	q_e_ (mg/g)	k_2_ (g/(mg∙min))	r^2^
0.109	1.795	0.939	0.117	22.667	0.983
Weber–Morris particle pore diffusion model parameters					
**Stage1**		**Stage2**		**Stage3**	
C_1_	k_1_	C_2_	k_2_	C_3_	k_3_
0.089	0.067	0.096	0.006	0.115	0.0004

**Table 2:** Freundlich and Langmuir isotherm model parameters.

T (K)	Langmuir Model			Freundlich Model		
	q_m_ (mg/g)	K_L_ (L/mg)	R^2^	K_F_	1/n	R^2^
298.15	24.379	0.614	0.987	7.859	0.441	0.974
308.15	31.969	0.364	0.968	8.234	0.494	0.912
318.15	43.695	0.289	0.996	9.416	0.545	0.985

**Table 3:** Comparison of adsorption saturation capacity with other adsorbents.

Adsorbent	Sb(III) Maximum Adsorption Capacity/(mg/g)	Reference
CHFS	24.37	This study
MNP@hematite	36.7	[46]
Iron-coated cork granulates	5.8	[36]
Cs functionalized iron nanosheet	138.8	[47]
Cu-doped Fe_3_O_4_	43.55	[48]
Fe_3_O_4_/GO	9.6	[49]
graphene	8.056	[50]
AC	2–3	[51]
C. sericea marine macroalgae	2.1	[52]
Green bean husk	20.1	[53]

**Table 4:** Thermodynamic model parameters.

Temperature (K)	∆G^0^ (kJ/mol)	∆H^0^ (kJ/mol)	∆S^0^ (kJ/(mol∙k))
298.15	−6.597	30.129	0.122
308.15	−7.248		
318.15	−9.047		

## Data Availability

The original contributions presented in the study are included in the article, further inquiries can be directed to the corresponding author.
